# Hepatic arterial embolization in patients with neuroendocrine tumors

**DOI:** 10.1186/1756-9966-33-43

**Published:** 2014-05-19

**Authors:** Michela Del Prete, Francesco Fiore, Roberta Modica, Vincenzo Marotta, Francesca Marciello, Valeria Ramundo, Antonella Di Sarno, Annachiara Carratù, Chiara de Luca di Roseto, Salvatore Tafuto, Fabiana Tatangelo, Robero Baldelli, Annamaria Colao, Antongiulio Faggiano

**Affiliations:** 1 Divisione di Endocrinologia, Dipartimento di Medicina Clinica e Chirurgia, Università di Napoli Federico II, Naples, Italy; 2 Divisione di Radiologia Interventistica, Dipartimento di Diagnostica per Immagini, Terapia Radiante e Metabolica, "Istituto Nazionale Tumori "Fondazione G. Pascale" - IRCCS, Naples, Italy; 3 Interventional Unit Ultrasound A.O. Dei Colli, Naples, Italy; 4 Divisione di Oncologia Medica Addominale, Dipartimento di Oncologia Addominale, "Istituto Nazionale Tumori "Fondazione G. Pascale" - IRCCS, Naples, Italy; 5 Divisione di Anatomia Patologica e Citopatologia, Dipartimento di Patologia diagnostica e di Laboratorio, "Istituto Nazionale Tumori "Fondazione G. Pascale" - IRCCS, Naples, Italy; 6 Divisione di Endocrinologia, Istituto Nazionale Tumori, Regina Elena, Rome, Italy

**Keywords:** Embolization, Chemoembolization, Liver metastases, Neuroendocrine tumor

## Abstract

Liver metastases occur in 46-93% of patients with neuroendocrine neoplasms (NENs). Presence and extension of liver metastases are considered important prognostic factors, as they may significantly impair the patient’s quality of life, because of either tumor bulk or hormonal hypersecretion. Therapies for NEN liver metastases include surgical resection, liver transplantation, chemotherapy and biotherapy. Surgery is the gold standard for curative therapy, but in most of NEN patients with liver metastases, when surgery can not be applied, minimally invasive therapeutic approaches are adopted. They include trans-arterial embolization (TAE), trans-arterial chemoembolization (TACE), radiofrequency thermal ablation and new emerging techniques.

TAE is based on selective infusion of particles in the branch of the hepatic artery supplying the tumor lesions. The goal of TAE is to occlude tumor blood vessels resulting in ischemia and necrosis. Many reports have shown that TAE can reduce tumor size and hormone output, resulting in palliation of symptoms without the use of cytotoxic drugs, resulting in better tolerability. This review will focus on TAE performance and safety in NEN patients with liver metastases.

## Introduction

Neuroendocrine neoplasms (NEN)s represent a heterogeneous group of neoplasms with distinct morphological and biological manifestations. According to the 2010 WHO classification, NENs are divided into: well differentiated neuroendocrine neoplasm (NEN) G1 (mitotic count <2 per 10 high power fields (HPF) and/or ≤2% Ki67 index), NEN G2 (mitotic count 2–20 per 10 HPF and/or 3–20% Ki67 index), and poorly differentiated high grade malignant neoplasm (NEC) G3 (mitotic count >20 per 10 116 HPF and/or >20% Ki67 index) [1]. Most common sites of origin are the gastrointestinal tract and the bronchopulmonary system. With a global incidence of approximately 5-7 cases per 100,000 per yr, gastroenteropancreatic NEN represents the second most frequent digestive cancer [2,3]. Metastatic involvement of the liver typically develops in about 46–93% of NEN patients [4,5]. In 12.9% of these patients, metastases are already detectable at the time of initial tumor diagnosis and 5-10% of them present with metastases and primary of unknown origin. Up to 75% of patients with small bowel NEN and 30-85% of those with pancreatic NEN present with liver metastases either at initial evaluation or during the course of their disease [6-8]. Presence and extension of liver metastases are considered important prognostic factors for NENs as they may significantly impair the patient’s quality of life because of either tumor bulk or hormonal hypersecretion. Liver metastases can result in a gradual replacement of liver parenchyma resulting in a progressive deficit of function until death, thus decreasing long term survival. Treatment of liver metastases can be curative or palliative. An effective treatment has to result in control of tumor growth and systemic hormonal effect, improvement of quality of life and increase of survival [9]. The treatment of liver metastases should take into account the natural history of the disease, the degree of liver involvement and the severity of related symptoms. The first line treatment of liver metastases is surgery and it can be curative for NEN G1/G2 or palliative. Complete resection (R0/R1) is associated with better long-term survival and quality of life. Resection of NEC G3 is not recommended, but may be considered in individual cases with isolated resecable metastases. Debulking resections (reduction of tumor mass >90%, resection of metastases and lymphnodes) can exceptionally be justified in palliative situations and incompleted debulking surgery (R2) has limited indication especially in functioning tumors [10]. However, only 10-20% of patients are eligible for either palliative or curative surgical resection. Liver transplantation is a potentially curative approach but limited to extremely selected patients and in experienced centers; moreover risk of recurrence persists in the transplantated liver [11].

For patients with multiple site metastases, systemic therapies are required to control tumor growth and clinical symptoms. They include chemotherapy (with streptozotocin or other agents), biotherapy with somatostatin analogs and/or alpha interferon and therapy with new agents targeting specific molecular pathways [12-17]. In most of NEN patients with liver metastases, minimally invasive, loco-regional approaches are adopted in place of surgery. They include trans-arterial embolization (TAE), trans-arterial chemoembolization (TACE), radiofrequency thermal ablation. Newly developed locoregional ablative procedures are under evaluation. TAE is based on selective infusion of particles in the branch (segmental or subsegmental) of the hepatic artery supplying the tumor lesions. The goal of TAE is to occlude tumor blood vessels resulting in ischemia and necrosis. TACE differs from TAE for the administration of a chemotherapeutic agent (anthracyclines such as Doxorubicin or Epirobicin) mixed with Lipiodol (fat-soluble contrast-medium with high concentration of Iodine; Lipiodol R), into the hepatic artery followed by the administration of embolizing agents (75-150 μm). In TAE treatment, Lipiodol administration (50%) is followed by the administration of embolizing agents (75-150 μm) without the administration of chemotherapeutic agents. Eligible patients for these procedures include NEN patients in metastatic phase, with predominant liver disease, which is judjed not resectable by surgery [18,19]. Although both techniques have been widely adopted, it remains debatable if the addition of cytotoxic drugs to embolization material increases the effectiveness of bland embolization alone, particularly when performed selectively [20,21]. This review will focus on TAE in NEN patients with liver metastases.

### Clinical, biochemical, instrumental characterization of NEN patients before TAE

Clinical work-up has to establish if the tumor is associated with a functioning endocrine syndrome which can result also in life-threatening conditions. Carcinoid syndrome is the most frequent functioning endocrine syndrome predominantly associated with the presence of liver metastases (60%). Regardless from endocrine symptoms, tumor mass-related symptoms need to be carefully evaluated, highlighting in particular the patient performance status, hepatic function and degree of liver involvement by the tumor, as liver metastases are often multilocular and bilateral [22]. Plasma chromogranin A (CgA) should be measured in all cases in order to have a potential sensitive marker, helpful for tumor monitoring and follow-up. However false-positive CgA false positive need to be carefully excluded [23,24]. The 24 h urinary 5-hydroxyindolacetic acid (5-HIAA) is an additional sensitive marker in NENs with carcinoid syndrome [25]. Other helpful NEN markers related to the specific syndrome are insulin, gastrin, glucagons or vasoactive intestinal polypeptide, to be evaluated according to the clinical picture [26,27].

Contrast-enhanced abdominal ultrasound and multidetector-row computed tomography (CT) are the standard initial imaging procedures. Advanced CT protocols and fusioning CT - positron emission tomography (PET) showed a sensitivity of 94–100% [28,29]. As an alternative to CT, magnetic resonance imaging (MRI), using gadolinium as liver-specific contrast agents, is recommended for routine staging, assessment of disease progression and monitoring of the therapy [30]. Functional imaging is mainly based on the [111-In-diethylene-triamine-penta-acetic-acid (DTPA)-D-Phe1]-octreotide (Octreoscan). Nowadays this technique has been replaced in several centers with ^68^Ga-radilabelled PET [31-33]. The diagnostic work-up of liver metastases should encompass tissue acquisition for histopathological and immunohistochemistry examination, since staging of NEN depends on markers of proliferation, such as Ki-67 and mitotic index and evaluation of vascular and neural invasiveness. Tumor staging predicts the prognosis and tailors the therapeutic strategy, particularly in patients who are not candidates for complete resection [34].

### Embolization procedures

Hepatic arterial embolization using a percutaneous Seldinger technique under radiological control was developed for metastatic endocrine tumors in the early 1970s. Indications for TAE generally include unresectability with symptoms related to tumor bulk, excessive hormone production, and rapid progression of liver disease. TAE has been shown to improve biophysical markers, palliate symptoms and reduce tumor burden at the radiological evaluation [20,35]. Neuroendocrine liver metastase are higly vascular and receive their blood supply from the hepatic artery (>90%), while normal liver receives 75-80% of its blood supply from the portal vein. TAE aims to create tumor ischemia embolizing the tumor feeding hepatic arterial branches [36]. Tumor ischemia has already been demonstrated useful in primary hepatocellular carcinoma, and now it finds indication for treatment of neuroendocrine liver metastases. In TACE procedure, tumor tissue ischemia is caused by both the chemotherapy activity and arterial embolization.

Different protocols have been used in TAE and embolizing agents are lipiodol, gel foam particles, polyvinyl alcohol (PVA) particles or microspheres [37]. Eligibility requirements included intact liver and renal function (bilirubin <2 mg/dL, serum creatinine level <2 mg/dL). Absolute contraindications were main portal vein occlusion and poor liver function. Other contraindications are: bilirubin greater than 2 mg/dL, hepatic tumor burden greater than 75%, specific contraindications to angiography such as allergy o contrast medium, fever and/or septic state, renal insufficiency, peripheral vascular disease, coagulopathies [38]. All patients were admitted to the hospital prior to the procedure and started intravenous hydration. Prior to embolization, a celiac angiogram was performed to identify the hepatic vasculature and ensure patency of the portal vein. Superior mesenteric artery angiogram was performed if needed to evaluate for accessory or replaced hepatic arteries supplying the liver. Embolization was performed until the selected vessel demonstrates complete or near complete stasis of flow. Usually the liver lobe with the bulkiest disease was embolized first. After embolization, patients were monitored in the hospital and discharged only after their liver enzymes had peaked. All patients were prophylactically administered antibiotics for one week in order to prevent abscess formation. Intravenous narcotics were typically administered for pain control. In case of recurrence or progression, TAE procedure can be performed several times [39]. When proximal embolization of tumor-feeding arteries in hepatic metastases was performed major effectiveness is remarked. Individual embolizations were spaced approximately 4 weeks apart and the majority of patients completed their embolizations in 2 or 3 times [9,40,41].

### Efficacy

Many reviews have been published on loco-regional ablative treatments of liver metastases of NENs. Several studies have been reported on TACE, while only few studies on TAE. This review focuses on TAE performance and safety in patients with liver metastases of NENs. It has to be highlighted that many authors did not report data on clinical response to TAE or reported these data as indirect consequence of decrease of tumour markers.

As a whole, 896 patients with NEN and liver metastases have been treated for a total of 979 TAE procedures. Median survival rates ranged from 10 to 80 months [9,21,35,39,42-52], but in the most of studies it was between 35 and 60 months (Table 1). Survival was reported to be correlated to objective tumor response. Progression free survival ranged from 0 to 60 months. Objective tumour response, including partial and complete response, was 50% as average (range, 2-100%). If we consider both tumour response and stabilization of tumor growth, the rate of patients who received a benefit from TAE was about 40% [9,21,35,39,42-52] (Table 1). Clinical response was about 56% (range, 9-100%). As far as biochemical response is concerned, TAE was reported to be effective in reducing biochemical markers in >50% of patients with NEN. In NEN patients with carcinoid syndrome, major decreases in 5-HIAA levels (>50% decrease as compared to baseline) occured in a range of 11-100% [9,35,39,42-44,51,53-57] (Table 2).

**Table 1:** Tumour response and survival rate in patients treated with Transarterial Embolization (TAE)

**Paper**	**Number and type of NEN**	**Number of TAEs**	**TR**	**OS**
**Loewe et al. 2003**[7]	23 carcinoids	75 TAE	4 (18%) CR, 12 (55%) PR, 6 (27%) PD	69 months
			(22 pts evaluable)	
**Gupta et al. 2003**[18]	69 carcinoids	Carcinoids:	Carcinoids: 46 (67%) PR, 6 (8.5%) MR, 11 (16%) SD, 6 (8.5%) PD	18 months
	54 PNENs	42 TAE/27 TACE	PNEN: 19 (35%) PR, 1 (2%) MR, 32 (59%) SD, 2 (4%) PD	
		PNENs:		
		32 TAE/22 TACE		
**Carrasco et al. 1986**[32]	25 carcinoids	25 TAE	20 (87%) CR, 1 (5%) PD	11 months
	(23 evaluable)			
**Strosberg et al. 2006**[36]	59 carcinoids	161 TAE	23 pts evaluable: 11 (48%) PR, 12 (52%) SD	36 months
	20 PNENs			
	5 unspecified NENs			
**Hanssen et al. 1989**[39]	19 carcinoids (7 evaluable)	7 TAE	7 (100%) PR	12 months
**Wangberg et al. 1996**[40]	64 carcinoids	40 TAE	---	60 months
**Eriksson et al. 1998**[41]	29 carcinoids	55 TAE	Carcinoids: 18 (62%) CR, 9 (31%) SD, 2 (7%) PD	80 months (carcinoids)
	12 PNENs		PNEN: 6 (67%) CR, 1 (11%) SD, 2 (22%) PD	20 months (PNEN)
**Brown et al. 1999**[42]	21 carcinoids	63 TAE	---	60 months
	14 PNENs			
**Chamberlain et al. 2000**[43]	41 carcinoids	59 TAE	33 pts evaluable: 19 (58%) SD	NR
	44 PNENs			
**Ruutiainen et al. 2007**[44]	67 unspecified NENs	23 TAE/44 TACE	(100%) CR	36 months
		(219 procedures)	(35%) CR	
**Ho et al. 2007**[45]	31 carcinoids	7 TAE/86 TACE	33 pts evaluable:	48 months
	15 PNEN		Carcinoids: 5 (23%) PR, 5 (23%) MR, 7 (31%) SD, 5 (23%) PD*	
			PNEN: 2 (18%) PR, 3 (27%) MR, 5 (46%) SD, 1 (9%) PD*	
**Kamat et al. 2008**[46]	60 unspecified NENs	33 TAE/27 TACE	12 (25%) PR, 6 (12%) MR, 22 (46%) SD, 8 (17%) PD*	9.3 months
		(123 procedures)	48 pts evaluable	
**Pitt et al. 2008**[47]	100 unspecified NENs	106 TAE/123 TACE	---	32.4 months
**Sward et al. 2009**[48]	107 carcinoids	213 TAE	---	56 months
**Fiore et al. 2014**[50]	12 PNENs	38 TAE/37 TACE	17 pts evaluable:	60 months
	16 NENs ileum		12 (70%) CR, 5 (30%) PR	
	2 NENs colon			

Legend = PNEN: NEN pancreas, TR: tumor response, OS: overall survival, PR: partial response, CR: complete response, MR: minor response, SD: stable disease, PD: progressive disease, NR: not reached, *cumulative results.

**Table 2:** Symptomatic and biochemical response in patients treated with TAE

**Paper**	**Number and type of NEN**	**Number of TAEs**	**BR**	**SR (endocrine symptoms)**	**SR (aspecific symptoms)**
**Loewe et al. 2003**[7]	23 small-bowel NENs	75	13 pts evaluable: 8 (61%) PR, 5 (39%) MR	9 pts evaluable:	- - -
				Abdominal pain 5 (56%) PR	
				Diarrhea 2 (22%) CR	
				Flushing 2 (22%) CR	
**Gupta et al. 2003**[18]	69 carcinoids	Carcinoids:	- - -	- - -	- - -
		42 TAE/27 TACE			
	54 PNENs	PNENs:			
		32 TAE/22 TACE			
**Carrasco et al. 1986**[32]	25 carcinoids	25	18 (72%) CR	- - -	20 (87%) CR
**Strosberg et al. 2006**[36]	59 carcinoids	161	35 pts evaluable:	Flushing and/or diarrhea 21 (48%) CR	9 (20%) CR
	20 PNENs		28 (80%) CR	Abdominal pain 11 (25%) CR	(44 pts evaluable)
	5 unspecified NENs		4 (11%) MR	Hypoglicemia 3 (7%) CR	
			3 (9%) no response	(44 pts evaluable)	
**Hanssen et al. 1989**[39]	19 carcinoids (7 pts evaluable)	7	7 (100%) PR	Diarrhea and/or flushing: 7 (100%) CR	- - -
**Wangberg et al. 1996**[40]	64 carcinoids	40	40 (100%) PR	- - -	40 (100%) PR
	(40 pts evaluable)				
**Eriksson et al. 1998**[41]	29 carcinoids	55	Carcinoids: 12 (41%) PR, 8 (28%) MR, 9 (31%) no response	- - -	11 carcinoid (38%) CR
	12 PNENs		PNEN: 6 (50%) PR, 2 (16%) MR, 4 (34%) no response		6 PNEN (50%) CR
**Brown et al. 1999**[42]	21 carcinoids	63	- - -	- - -	46 (96%) PR
	14 PNENs	(48 evaluable)			(48 TAE evaluable)
**Chamberlain et al. 2000**[43]	41 carcinoids	59	- - -	33 pts evaluable	31 (94%) PR
	26 non functional PNENs			Hormonal and/or pain symptoms	
	18 functional PNENs			31 (94%) PR	
**Ruutiainen et al. 2007**[44]	67 unspecified NENs	23 TAE/44 TACE (219 procedures)	- - -	- - -	- - -
**Ho et al. 2007**[45]	46 NENs	7TAE/86 TACE	- - -	- - -	27 pts evaluable
	(31 carcinoids; 15 PNEN)				21 (78%) PR
**Kamat et al. 2008**[46]	60 unspecified NENs	33 TAE/27 TACE	- - -	- - -	20 pts evaluable
		(123 procedures)			13 (65%) PR
**Pitt et al. 2008**[47]	100 unspecified NENs	106TAE/123TACE	- - -	- - -	35 pts evaluable: 29 TAE (83%) PR
					35 pts evaluable: 32 TACE (86%) PR
**Sward et al. 2009**[48]	107 carcinoids	213	37 pts evaluable:	Diarrhea and/or flushing 76 (71%) CR	76 (71%)
			CgA: 19 (51%) CR		
			54 pts evaluable:		
			5HIAA: 26 (48%) CR		
**Fiore et al. 22014**[50]	12 PNENs	38 TAE/37 TACE	- - -	- - -	19 pts evaluable
	16 NENs ileum				(64%) PR*
	2 NENs colon				

Legend = PNEN: NEN pancreas, BR: biochemical response, SR: symptomatic response, PR: partial response, CR: complete response, MR: minor response.

*Cumulative results.

The first study reporting on TAE treatment in patients with liver metastases from NEN was published by Carrasco et al. [35]. A response to TAE was observed in 95% of patients with malignat liver metastases from carcinoids, with a median response duration of 11 months. Tumour response was subsequently confirmed in all studies performed on TAE and the rate of patients responsive to treatment (objective response plus stability) was always about or more than 80% and the median reponse duration was about 36 months [9,21,39,47-49,52] (Table 1).

In the Carrasco study, a symptomatic response occurred in 87% of patients and correlated with size decrease of liver lesions. In the Fiore study a symptomatic response occurred in 64% of patients who had an uncontrolled endocrine syndrome [52]. Furthermore, a decrease in urine 5-HIAA concentrations of about 41% as average has been reported [35]. A similar o greater effect on 5-HIAA was confirmed in subsequent studies [9,35,39,42,43,51,52] (Table 2). When combined with somatostatin analogs or interferon therapy, TAE was found to be still more effective in reducing 5-HIAA and controlling carcinoid syndrome [42,43] (Table 2). The biochemical response to repeated TAE cycles was similar to that observed after the first cycle. Finally, the biochemical response was also found to be correlated with survival [51] (Table 2).

Some studies reported a comparison between carcinoid tumors (according to old classifications of NEN) and pancreatic NENs. Eriksson et al. reported a median survival of 80 months in patients with midgut carcinoid tumors and 20 months in those with pancreatic NENs [42] (Table 1). Similar difference was reported in the Gupta study where progression free survival as well as tumor response rate were higher in carcinoids than in pNENs [21]. On the contrary, no difference in overall survival, progression free survival and objective response was reported by Ho et al. [48] (Table 1).

On the other hand, symptomatic response and duration of the response were similar for patients with carcinoid tumors and pancreatic NEN [21,35,42-46,48,51,52] (Table 2). In general, the duration of response was longer in patients treated for hormonal symptoms with or without pain, while it was shorter when the indication was pain alone [45] (Table 2).

### Safety

TAE was found to be a quite safe procedure. Range of TAE-related death was from 2 to 13 patients, with a total of 50 deaths. Adverse events such as ischemia of biliary tree, post-embolization syndrome may occur. Complications were observed in a total of 125 patients (14%) of all 896 patients with NENs, but it is not always clarified wether adverse events and toxicity occurred after TAE and/or TACE (Table 3). Post-embolization syndrome includes abdominal pain, nausea, fevers, hypertension, thrombocytopenia, leukocytosis, transient increase in liver enzymes (predominantly transaminases) and LDH which generally comes down within a few days to 2-3 weeks. Increased bilirubin levels have also been noted. Ischemia of the biliary tree has also been rarely reported and moderate elevation of alkaline phosphatase. When some devices were considered to keep the patient well hydrated and in supportive care, post-embolization syndrome resulted to be less frequent [4,44]. As a whole, TAE may be considered a quite safe procedure, given the high number of procedures carried out (979) and the low number of deaths (50 patients: 6%) and complications (125 patients: 14%) (Table 3).

**Table 3:** Safety of TAE

**Paper**	**Number and type of NEN**	**Number of TAE**	**Complications**	**Death**
**Loewe et al. 2003**[7]	23 small-bowel NENs	75	Decreased body weight 1 (1%)	2 (8%)
			Leg pain 1 (1%)	
**Gupta et al. 2003**[18]	69 carcinoids	Carcinoids:	Serious adverse events 19 (15%)*	1 (1%)
	54 PNENs	42 TAE/27 TACE		
		PNENs:		
		32 TAE/22 TACE		
**Carrasco et al. 1986**[32]	25 carcinoids	25	- - -	2 (8%)
	(23 evaluable)			
**Strosberg et al. 2006**[36]	59 carcinoids	161	- - -	2 (2%)
	20 PNENs			
	5 unspecified NENs			
**Hanssen et al. 1989**[39]	19 carcinoids	7	- - -	- - -
	(7 evaluable)			
**Wangberg et al. 1996**[40]	64 carcinoids	40	- - -	Increased risk of cardiovascular deaths (not specified)
**Eriksson et al. 1998**[41]	29 carcinoids	55	Unspecified severe complications 6 (10%)	13 (31%)
	12 PNENs			
**Brown et al. 1999**[42]	21 carcinoids	63	Unspecified severe complications 11 (17%)	4 (6%)
	14 PNENs			
**Chamberlain et al. 2000**[43]	41 carcinoids	59	- - -	4 (6%)
	26 non functional PNENs			
	18 functional PNENs			
**Ruutiainen et al. 2007**[44]	67 unspecified NENs	23 TAE/44 TACE	Unspecified toxicity 34 (50%)*	(1) 1.4%*
		(219 procedures)		
**Ho et al. 2007**[45]	46 NENs	7 TAE/86 TACE	Unspecified complications 9 (10%)*	4 (4.3%)
	(31 carcinoids; 15 PNEN)			
**Kamat et al. 2008**[46]	60 unspecified NENs	33 TAE/27 TACE	Unspecified complications 21 (35%)*	12 (20%)
		(123 procedures)		
**Pitt et al. 2008**[47]	100 unspecified NENs	106TAE/123TACE	Liver abscesses, ileus, groin hematoma, hypotension 7 (13%) TAE hematoma, acute renal failure, and a biloma 3 (6%) TACE	3 (3%)*
**Sward et al. 2009**[48]	107 carcinoids	213	Liver abscess 4 (4%)	2 (2%)
			Mild pancreatitis 1 (1%)	
			Accidental occlusions of the common hepatic artery 2 (2%)	
**Fiore et al. 2014**[50]	12 PNENs	38 TAE/37 TACE	Post-embolization syndrome 6 (40%) TAE	0%
	16 NENs ileum		Post-embolization syndrome 8 (60%) TACE	
	2 NENs colon			

*Cumulative results.

## Conclusions

TAE appears to be an optimal treatment approach for inoperable liver metastases from NENs, for higher metastatic load, for management of symptoms alone and in association with interferon or somatostatin analogues, suggesting a prolonged 5-yr survival and local tumor control and for survival improvement [42,43,45,51]. Tumor response as well as survival, but not clinical and biochemical response, appear to be better for patients with carcinoid than pancreatic NENs.

TAE is considered a safe procedure. The low number of complications during and/or after TAE procedures can be easily and quickly treated, while the small number of deaths further confirms the safety of this technique. Moreover the deaths are often associated with adverse effects not related to TAE, but with the chemotherapeutic agents used for TACE. It is essential that TAE is performed by highly qualified and specialized team. Finally, the presence of extra-hepatic metastases or unresected primary tumor should not limit the use of TAE [48] since the liver function plays the most important role in the survival of these patients.

On the other hand, TAE should be avoided in patients with massive tumor burden and severely compromised liver function, poor performance status, sepsis, carcinoid heart disease and other risk factors for treatment related mortality (Table 4). In these cases less aggressive TAE, repeated if needed, can be effective, while decreasing the risk for procedure related mortality [49,50].

**Table 4:** Indications and contraindications of TAE in patients with NENs

**Indications**	**Contraindications**
- NEN tumor functioning or not	- Massive tumor burden
- Highly vascularised liver metastases	- Severely compromised liver function
- Liver metastases >3 in number and or >3 cm in size	- Poor performance status
	- Sepsis
- Patients with tumor mass-related symptoms and/or carcinoid syndrome	- Carcinoid heart disease and other risk factors for treatment related mortality

Future randomized, prospective clinical trials comparing safety, efficacy and lorng term outcomes of different treatment approaches for liver metastases in NEN patients with comparable disease, should better define the role of TAE. In conclusion, available data suggest TAE as a safe therapeutic option in patiens with liver metastases from NENs, effective for controlling tumor progression and improving mass and endocrine symptoms, while increasing long term survival. In order to minimize risk related procedure TAE should be performed in a multidisciplinary setting and in experienced NEN centers. Finally, the choice of TAE instead of TACE, PRRT, chemotherapy or biotherapy should be performed in a multidisciplinary setting and in experienced NEN centers, according to patient and tumor characteristics.

## Abbreviations

NENs: Neuroendocrine neoplasms; TAE: Trans-arterial embolization; TACE: Trans-arterial chemoembolization; CgA: Chromogranin A; 5-HIAA: Urinary 5-hydroxyindoleacetic acid; CT: Computed tomography; PET: Positron emission tomography; MRI: Magnetic resonance imaging; Octreoscan: [111-In-diethylene-triamine-penta-acetic-acid (DTPA)-D-Phe1]-octreotide; DOTA: Tricarboxy-methyl-1-yl-acetyl-D-Phe1; DOTATOC: Try3–octreotide; DOTANOC: DOTA-1-Nal3-octreotide; PVA: Polyvinyl alcohol; PNEN: Neuroendocrine neoplasm of pancreas; TR: Tumor response; OS: Overall survival; PR: Partial response; CR: Complete response; MR: Minor response; SD: Stable disease; PD: Progressive disease; BR: Biochemical response; SR: Symptomatic response.

## Competing interest

All the authors declare that there are no competing interest that could be perceived as prejudicing the impartiality of the data reported.

## Authors' contribution

All the authors contributed to the preparation of this review. MDP and FF wrote the review, RM, VM, FM, VR, ADS, ST, FT and RB contributed to the research of articles of literature, ACC and CC LDR made the tables and finally AC and AF wrote and revised the review. All authors read and approved the final manuscript.
